# *Porcine circovirus 2* (PCV-2) genotype update and proposal of a new genotyping methodology

**DOI:** 10.1371/journal.pone.0208585

**Published:** 2018-12-06

**Authors:** Giovanni Franzo, Joaquim Segalés

**Affiliations:** 1 Department of Animal Medicine, Production and Health, University of Padova, Legnaro, Italy; 2 UAB, Centre de Recerca en Sanitat Animal (CRESA, IRTA-UAB), Campus de la Universitat Autònoma de Barcelona, Bellaterra, Spain; 3 Departament de Sanitat i Anatomia Animals, Facultat de Veterinària, Universitat Autònoma de Barcelona, Bellaterra Spain; Institute for Integrative Biology of the Cell, FRANCE

## Abstract

*Porcine circovirus 2* (PCV-2) is one of the most widespread viral infections of swine, causing a remarkable economic impact because of direct losses and indirect costs for its control. As other ssDNA viruses, PCV-2 is characterized by a high evolutionary rate, leading to the emergence of a plethora of variants with different biological and epidemiological features. Over time, several attempts have been made to organize PCV-2 genetic heterogeneity in recognized genotypes. This categorization has clearly simplified the epidemiological investigations, allowing to identify different spatial and temporal patterns among genotypes. Additionally, variable virulence and vaccine effectiveness have also been hypothesized. However, the rapid increase in sequencing activity, coupled with the *per se* high viral variability, has challenged the previously established nomenclature, leading to the definition of several study-specific genotypes and hindering the capability of performing comparable epidemiological studies.

Based on these premises, an updated classification scheme is herein reported. Recognizing the impossibility of defining a clear inter-cluster p-distance cut-off, the present study proposes a phylogeny-grounded genotype definition based on three criteria: maximum intra-genotype p-distance of 13% (calculated on the ORF2 gene), bootstrap support at the corresponding internal node higher than 70% and at least 15 available sequences. This scheme allowed defining 8 genotypes (PCV-2a to PCV-2h), which six of those had been previously proposed. To minimize the inconvenience of implementing a new classification, the most common names already adopted have been maintained when possible. The analysis of sequence-associated metadata highlighted a highly unbalanced sequencing activity in terms of geographical, host and temporal distribution. The PCV-2 molecular epidemiology scenario appears therefore characterized by a severe bias that could lead to spurious associations between genetic and epidemiological/biological viral features. While the suggested classification can establish a “common language” for future studies, further efforts should be paid to achieve a more homogeneous and informative representation of the PCV-2 global scenario.

## Introduction

Initial sequence analyses of different *Porcine circovirus 2* (PCV-2) viruses around the world indicated a close phylogenetic relationship, with a nucleotide sequence identity higher than 93% [1]. Although genetic differences among strains/sequences of PCV-2 were considered minimal, when a nucleotide diversity cut-off of 3.5% was applied to existing sequences, they could be divided into two major phylogenetic groups. Subsequently, those genetic groups were called genotypes; after a number of nomenclature proposals for these genotypes by different research groups, a consensus (genotypes “a” and “b”) was reached [2]. Subsequently, a third genotype was retrospectively reported from Denmark in the 1980s and named PCV-2c [3]. Recent data indicated the current existence of this genotype in feral pigs from Brazil [4] and in domestic swine in China [5]. A fourth genotype named PCV-2d was lately described [6], although retrospectively detected in Switzerland already in 1998 [7]. In fact, it was initially called as a mutant of PCV-2b (mPCV-2b) and linked to potential vaccination failure cases [8]. Further studies have shown that PCV-2d is widely spread all over the world nowadays, becoming more and more prevalent. More recently, a new genotype (PCV-2e) has been identified in swine samples from USA and Mexico [9,10]. In addition, a Chinese study found a year ago novel viral sequences clustering differently from the existing ones and tentatively classified them as genotype PCV-2f [11]. Currently, it is considered that PCV-2d exhibits similar virulence to PCV-2a and PCV-2b when inoculated into susceptible pigs [12], but the clinical significance of PCV-2c, PCV-2e and PCV-2f are not known. Certainly, the appearance of new genotypes in the future is likely, since PCV-2 is a single stranded DNA virus with high nucleotide substitution rate (comparable to those of RNA viruses) and the possibility to generate mutations of the genome is high [13].

Recent phylodynamic studies have indicated that PCV-2 population dynamics have been characterized by the appearance of periodic waves of different genotypes which, after an initial increase in prevalence, spread following major swine commercial routes and were then superseded by subsequent emerging genotypes [14]. PCV-2a was the most prevalent genotype in clinically affected pigs from 1996 to the early 2000s, after which PCV-2b predominated (“genotype shift”). The emergence of PCV-2b in North America and Europe was associated with the appearance of a more severe clinical disease [15–18]. PCV-2d was first reported in China [6] and a second “genotype shift” (from PCV-2b to PCV-2d) seems to be occurring globally [7,14], perhaps driven by the worldwide use of PCV-2 vaccines.

Importantly, different PCV-2 strains of the same or different genotype can be detected in the same pig [19]. Also, *in vivo* and *in vitro* studies have provided evidence of potential viral recombination. In fact, some circulating recombinant forms (named as CRFs) have followed comparable population dynamics and spreading routes to those of well-defined genotypes, suggesting that recombinant strains are able to circulate and compete with parental ones [14].

Taking into account the very recent description of novel PCV-2 genotypes and the growing difficulty for classifying consistently current and novel viral sequences based on initially proposed methods, this work aimed to review existing classification criteria and propose a further developed method for PCV-2 genotyping.

## Material and methods

### Sequence dataset

All available PCV-2 sequences were downloaded from GenBank (https://www.ncbi.nlm.nih.gov/genbank/) using the species-specific taxonomic identifier code (i.e. 85708) and records whose complete ORF2 gene was available were selected. All sequences were aligned at codon level using the MAFFT method implemented in TranslatorX [20], and the obtained alignment was inspected both visually and using dedicated Python scripts. Poorly aligned sequences, those displaying premature stop codons, frameshift mutations or unknown bases, suggestive of poor sequence quality, were removed. Alignment was then performed again on the refined dataset.

### Recombination and phylogenetic analyses

To reduce the computational burden and remove data redundancy, only one sequence representative of all identical ones was identified using ElimDupes (https://www.hiv.lanl.gov/content/sequence/ELIMDUPES/elimdupes.html) and maintained for further analyses.

Recombination analysis was performed on the non-redundant ORF2 sequences dataset using RDP4 v4.91 [21]. The RDP, GENECONV and MaxChi methods were selected as primary scan, while all the methods implemented in RDP4 were used for recombination detection refinement. For each method, settings were adjusted considering the database features according to the software manual recommendations. A recombination event was accepted if detected by more than two methods with a significance p-value of p < 0.01 with Bonferroni’s correction. All sequences identified as recombinants where excluded from further analysis.

Pairwise genetic p-distance was calculated for all sequences using the *ape* version 5.2 [22] package in R version 3.3.2 [23] in order to graphically evaluate, by means of a density plot, the presence of reasonable inter-group separation cut-off.

A Neighbor-Joining phylogenetic tree based on raw genetic distances (pairwise p-distance) was reconstructed using MEGA7 [24]. The reliability of the sequence cluster was evaluated by performing 1000 bootstrap replicates.

The new proposed genotype definition was based on within-cluster genetic distance and clade reliability, defined by bootstrap support. Several arbitrary genetic distances thresholds were attempted to define a reasonable cluster number, maintaining the correspondence with the existing PCV-2 genetic diversity (genotypes defined to date). As final setting, ClusterPick [25] was used to identify sequence groups featured by a within-cluster genetic distance lower than 13% and bootstrap support of the relative ancestral node higher than 70%.

### Genotype temporal, geographical and host distribution

The non-redundant database was expanded to its original size, and the accession numbers of sequences classified within each cluster were recorded. Collection country, host and date were downloaded from GenBank using homemade python scripts benefiting of the functions of the Biophyton library [26]. To facilitate data analysis and graphical representation, aggregated categories were created (e.g., countries were aggregated to continent level, similar host were merged, etc.). To assess the presence of statistically significant differences in the cluster distribution among categories, a Chi-square or Fisher test was performed. The presence of over- or under represented cluster-category pairs was assessed by evaluating the respective z-score. Statistical significance was set to p-value < 0.05.

## Results

A total of 6,171 PCV-2 sequences were downloaded from GenBank (accessed 05-July-2018). However, after database refinement and potential recombinant sequence removal (about 22% of the initial sequence number), 4,586 ORF2 sequences were maintained in the analysis, corresponding to 2,610 unique sequences.

**Fig 1:**
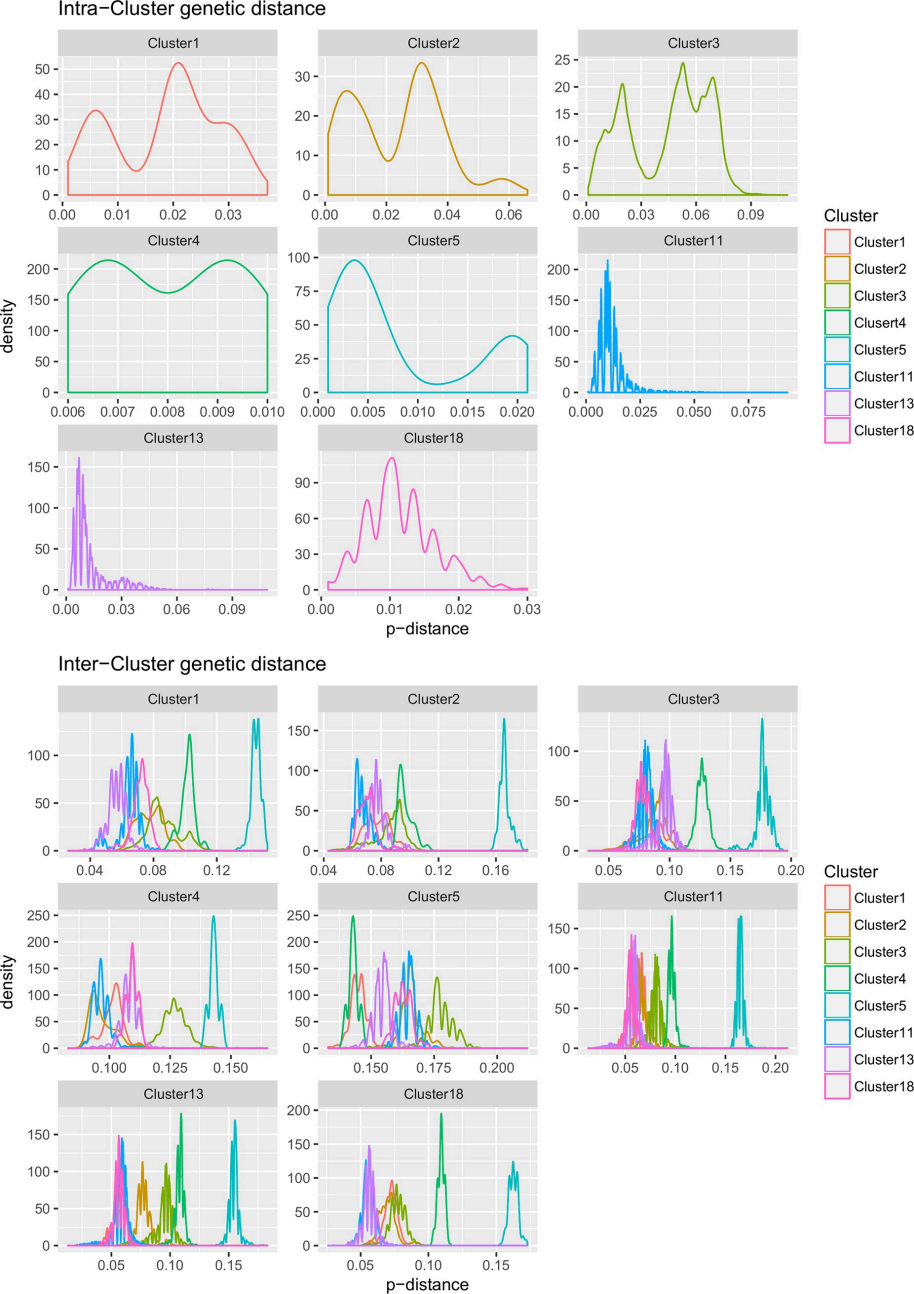
Intra and inter-cluster pairwise p-distances among ORF2 sequences. Density plot reporting the pairwise p-distance distribution within and between clusters. Different clusters have been color-coded.

Analysis of p-distance profile distribution evidenced the absence of any clear cut-off to divide PCV-2 genetic variability in a limited, but still meaningful, genotype number (Fig 1). Therefore, the analysis of phylogenetic clusters was preferred instead.

Cluster feature analysis led to the definition of 18 genotypes (S1 Table). However, only 7 clusters (Cluster1, Cluster2, Cluster3, Cluster5, Cluster11, Cluster13, and Cluster18) included more than 15 sequences in the original dataset and were considered for being potentially recognized as genotypes and for further analyses. A summary of the sequence number classified in each cluster/genotype is reported in Table 1.

**Table 1:** Count of sequences classified in each cluster/genotype-continent pair. The respective column percentage is reported between brackets. For an easier interpretation, only major clusters corresponding to proposed genotypes are reported.

Cluster	Genotype	Africa	Asia	Europe	North America	Oceania	South America	Total
**Cluster3**	PCV-2a	1 (100%)	299 (12.6%)	50 (10,39%)	301 (22.69%)	8 (80%)	16 (11.67%)	675 (15,63%)
**Cluster11**	PCV-2b	0 (0%)	886 (37.51%)	374 (77,75%)	614 (46.30%)	2 (20%)	108 (78.83%)	1984 (45,94%)
**Cluster4**	PCV-2c	0 (0%)	0 (0%)	3 (0,62%)	0 (0%)	0 (0%)	1 (0,724)	4 (0,09%)
**Cluster13**	PCV-2d	0 (0%)	1065 (45.08%)	41 (8,52%)	373 (28.13%)	0 (0%)	12 (8.75%)	1491 (34,52%)
**Cluster5**	PCV-2e	0 (0%)	2 (0.08%)	0 (0%)	20 (1.50%)	0 (0%)	0 (0%)	22 (0,50%)
**Cluster2**	PCV-2f	0 (0%)	25 (1.05%)	1 (0,20%)	9 (0.67%)	0 (0%)	1 (0.72%)	36 (0,83%)
**Cluster1**	PCV-2g	0 (0%)	10 (0.42%)	12 (2,49%)	9 (0.67%)	0 (0%)	0 (0%)	31 (0,71%)
**Cluster18**	PCV-2h	0 (0%)	75 (3.17%)	0 (0%)	0 (0%)	0 (0%)	0 (0%)	75 (1,73%)
**Total**		1 (100%)	2362 (100%)	481 (100%)	1326 (100%)	11 (100%)	137 (100%)	4318 (100%)

**Fig 2:**
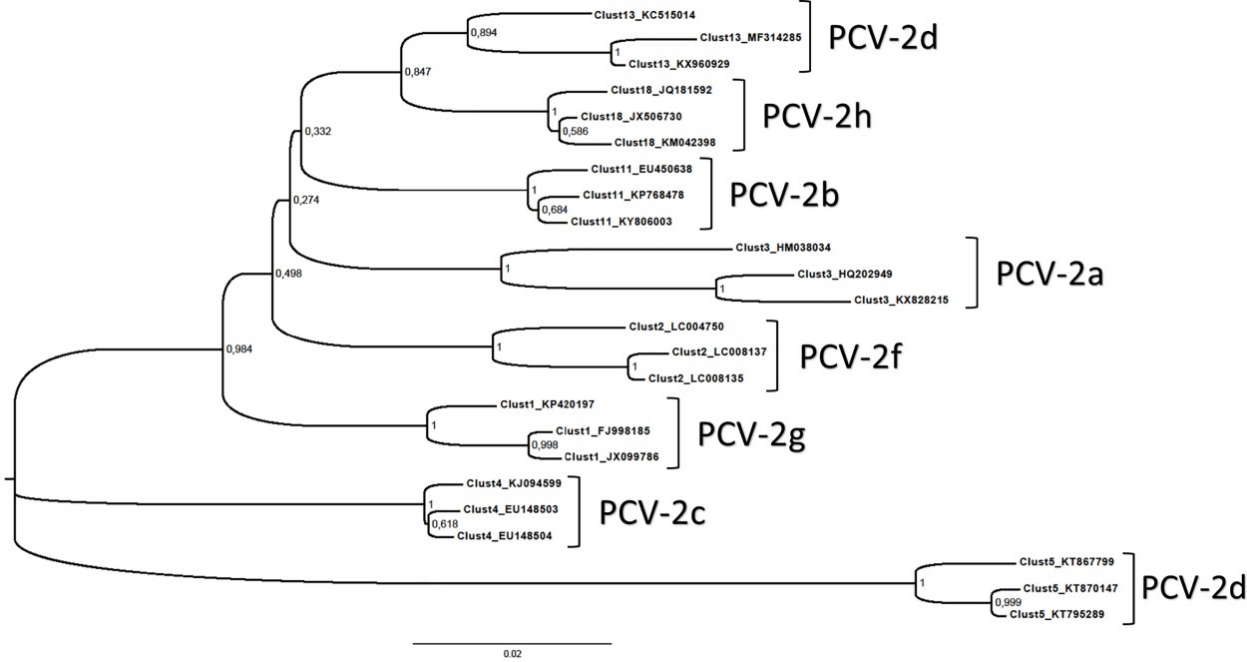
PCV-2 Neighbor-Joining phylogenetic tree. Neighbor-Joining phylogenetic tree reconstructed based on row genetic distances (i.e. pairwise p-distance) calculated on a collection of strains representative of the proposed PCV-2 genotypes. Both the cluster and genotype nomenclature are reported. Bootstrap support is displayed near the corresponding node. A tree reconstructed based on a more extensive sequence collection (n = 2610) is provided as S1 Fig.

Some of the defined clusters mirrored previously defined genotypes (i.e. Cluster2 = PCV-2f; Cluster3 = PCV-2a; Cluster 4 = PCV-2c (maintained for historical reasons); Cluster 5 = PCV-2e; Cluster11 = PCV-2b; and Cluster13 = PCV-2d). Additionally, two more genotypes are proposed (Cluster1 = PCV-2g; Cluster18 = PCV-2h) (Fig 2).

**Fig 3:**
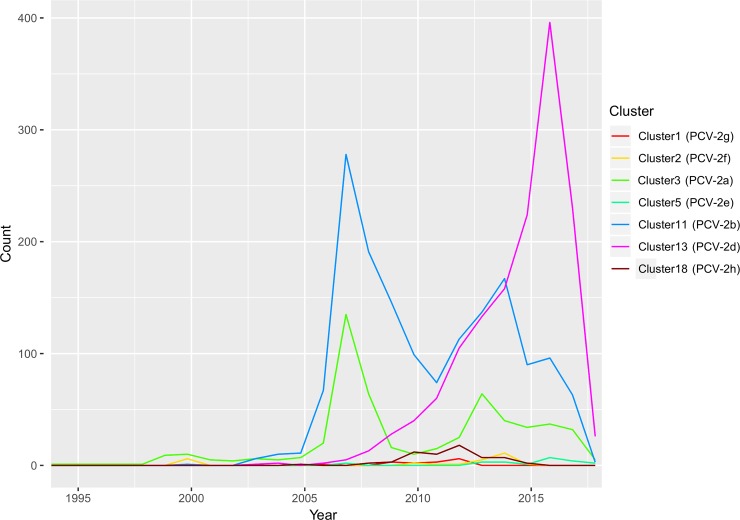
Count of sequences for each collection year. Different clusters have been color-coded.

**Fig 4:**
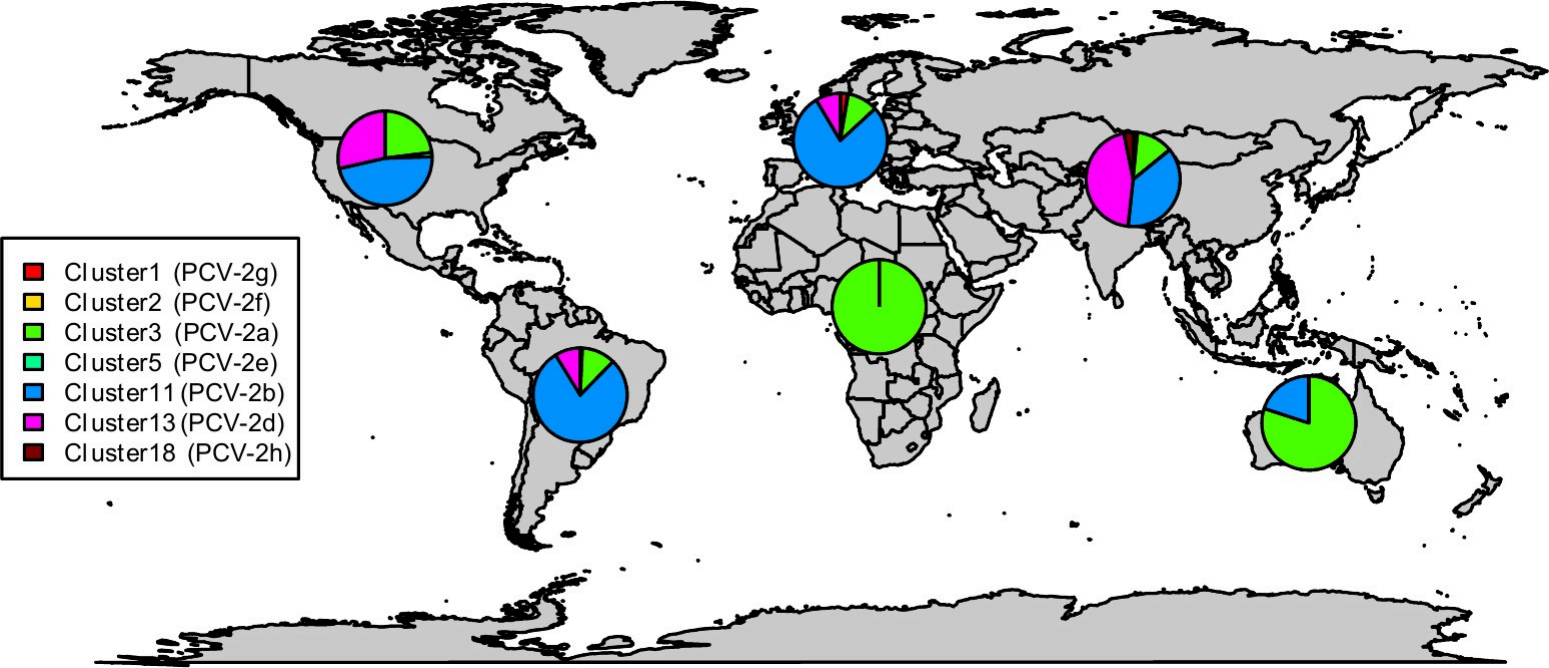
Geographic distribution of major PCV-2 clusters. The PCV-2 cluster distribution has been summarized using continent-specific pie-charts whose slide size is proportional to the cluster frequency (color-coded).

Among all these groupings, Cluster11 (PCV-2b) included the highest sequence number, followed by Cluster13 (PCV-2d) and Cluster3 (PCV-2a). Nevertheless, a clear temporal trend was evident, being the Cluster3 the first one with a relevant number of published sequences, followed by Cluster 11 (especially from 2005 onwards) and, finally, Cluster13, which demonstrated a progressive increase in sequenced strains over time (Fig 3). Cluster3, 11 and 13 displayed an actual worldwide distribution, being detected in all continents with the exception of Africa and Oceania, for which the number of available sequences was negligible (Table 1 and Fig 4). Cluster3 was the most prevalent one in Africa and Oceania, Cluster11 in America and Europe and Cluster13 in Asia (Fig 4). A non-random distribution was observed in PCV-2 cluster geographical pattern (p<0.001).

The vast majority of available sequences were collected from domestic pig (*Sus scrofa domesticus*). In detail, among the recognized genotypes, 3,386 out of 3,555 (95.2%) sequences, for which collection host was known, originated from domestic pig, 155 (4.36%) from wild boar, 11 (0.31%) from ruminants, 2 (0.056%) from human beings and 1 from *Rattus norvegicus* (0.028%). A summary of Host-Cluster association is reported in Table 2.

**Table 2:** Count of sequences classified in each cluster/genotype-host pair. The respective column percentage is reported between brackets. For interpretation easiness, only major clusters corresponding to proposed genotypes are reported.

.	Genotype	*Homo sapiens*	*Rattus norvegicus*	Ruminant	Domestic pig	Wild boar	Total
**Cluster3**	PCV-2a	1 (50%)	0 (0%)	2 (18.18%)	500 (14,74%)	17 (10.96%)	520 (14,61%)
**Cluster11**	PCV-2b	1 (50%)	1 (100%)	6 (54.54%)	1447 (42,68%)	114 (73.54%)	1569 (44,08%)
**Cluster4**	PCV-2c	0 (0%)	0 (0%)	0 (0%)	4 (0,11%)	0 (0%)	4 (0,11%)
**Cluster13**	PCV-2d	0 (0%)	0 (0%)	3 (27.27%)	1318 (38,88%)	11 (7.09%)	1332 (37,42%)
**Cluster5**	PCV-2e2e	0 (0%)	0 (0%)	0 (0%)	22 (0,64%)	0 (0%)	22 (0,62%)
**Cluster2**	PCV-2f	0 (0%)	0 (0%)	0 (0%)	26 (0,76%)	1 (0.64%)	27 (0,75%)
**Cluster1**	PCV-2g	0 (0%)	0 (0%)	0 (0%)	8 (0,23%)	12 (7.74%)	20 (0,56%)
**Cluster18**	PCV-2h	0 (0%)	0 (0%)	0 (0%)	65 (1,917%)	0 (0%)	65 (1,826%)
**Totale**		2 (100%)	1 (100%)	11 (100%)	3390 (100%)	155 (100%)	3559 (100%)

A Chi-square test performed on the more represented clusters and hosts demonstrated a non-random association (p<0.001). Particularly, Cluster1 and 11 were overrepresented in wild boar, although this might be attributable to the low number of available sequences. On the other hand, *Sus scrofa domesticus* was under-represented in Cluster1 and wild boar in Cluster13 (Table 2 and S2 Fig).

To facilitate PCV-2 classification in routine settings, a dataset of reference sequences, annotated with the corresponding cluster/genotype name, has been provided (S1 Table).

## Discussion

Extensive genetic studies on PCV-2 have proven the high heterogeneity of this pathogen, which can be attributed to both high mutation and recombination rates [13,14]. Interestingly, PCV-2 variability has been potentially linked to different phenomena with remarkable practical implications, like differential epidemiological fitness [14], virulence [27], cross-protection [28], vaccine escape [29], etc., although definitive association has not consistently been proven in most instances because of limited data availability and poor results reproducibility. The multi-factorial nature of porcine circovirus diseases [30] can conceal the identification of causal nexus in absence of consistent and properly collected information. This scenario highlights the need for continuous, massive and updated epidemiological monitoring to investigate and understand PCV-2 epidemiological features and determinants. To this purpose, a classification scheme that simplifies and summarizes the peculiarities of each strain is mandatory for both routine diagnosis and viral characterization as well as for more complex association studies with important facts (virulence and cross-protection, mainly).

Currently, the most widely accepted classification scheme defines 4 genotypes identified based on phylogenetic analysis [31]. Since its publication, additional genetic groups have been described all around the world and claimed as new genotypes [9–11]. Nevertheless, in most instances, the newly detected strains were compared to a limited reference number, potentially magnifying some differences or missing the presence of additional inter-cluster relationships. Therefore, the present study proposes a new and updated genotype classification scheme based on a wide collection of ORF2 sequences, including all freely available non-recombinant ones. This particular region was selected since the *cap* gene is considered the more suitable phylogenetic and epidemiological marker for PCV2 and is able to reconstruct the same tree as the whole viral genome [1].

It must be stressed that, as already recognized in the previous classification scheme [31], no clear genetic distance cut-off could be identified to separate PCV-2 groups, since a relevant overlap between intra- and inter-genotype p-distance is present (Fig 1). Consequently, the proposed classification is substantially based on the phylogenetic relationship among strains, with adequate bootstrap support.

With the aim of providing a genotype definition able to depict the PCV-2 genetic heterogeneity, allowing at the same time a practical application in routine diagnostic and epidemiological studies (avoiding too many groupings and sub-groupings), it is suggested to limit the genotype definition to the following criteria: maximum within genotype p-distance of 13%, minimum cluster internal node bootstrap support of 70% and at least 15 identified sequences (A workflow of the proposed genotype analysis is depicted in S3 Fig). The last criterion was imposed to focus on more widespread genotypes and to avoid the risk of defining poor quality sequences as separate genotypes. An exception to this classification scheme is PCV-2c, which was maintained as a recognized genotype because of its historical description [3–5], although not fulfilling the third criterion yet.

The suggested classification is based on a NJ tree, reconstructed using raw genetic distances (i.e. pairwise p-distance). Certainly, other approaches like maximum likelihood, with more complex substitution models and rate heterogeneity among sites can better represent the phylogenetic relationship among strains. However, these methods imply a heuristic search in the tree likelihood space and estimation of all model parameters, being both database-dependent. In other words, somewhat different estimates (and thus different tree topology and branch lengths) can be obtained depending on the sequences included in the dataset. Since the present study aim was to establish a robust and long lasting approach for genotype definition, a simpler approach, less susceptible to future (and desirable) database updates, was selected. Overall, albeit all the previously proposed genotypes have been confirmed with the new methodology, other divergent genetic groups were discovered, demonstrating a higher variability than that previously expected for PCV-2.

Although the increase in the number of genotypes may be potentially surprising, these results could have been predicted for several reasons. The progressive increase in sequence number, obtained from previously non-sampled countries, could have revealed a hitherto undetected genetic variability. Additionally, the inclusion of thousands of sequences in the same analysis (instead of a limited set of them) understandably increases the capability of depicting the overall PCV-2 variability picture and identifying previously neglected genetic groups. Remarkably, most of the sequences included in newly defined genotypes were marked as “direct submission” in GenBank. Thus, although a relevant subset of those strains was sequenced years ago, most of them were not properly analyzed. The relevance of periodic and systematic analysis of freely available molecular data is therefore highly recommended to recognize the emerging genetic variability promptly.

The evolution of viruses with a high mutation rate genome is not expectable to follow a limited number of directions and a tendency to explore several paths in the fitness landscape can be predicted [32–34]. Accordingly, only 7 out of 18 recognized clusters/genotypes included more than 15 sequences and only 3 were consistently detected for the whole considered time period and in substantially all continents. Therefore, a relevant proportion of the detected heterogeneity could be attributed to epidemiological dead ends, which underwent extinguished or circulated in extremely reduced host populations. The well-known history of PCV-2c (here named Cluster4) represents a good example in this sense [3,4].

Some of the clusters (i.e., Cluster1 and Cluster18) included a subset of sequences proposed as potential recombinant strains by some (but not all) previous studies [35,36]. The inclusion of hundreds of additional sequences could have affected the recombination detection in several ways. The sequencing of new strains, representative of “intermediate steps” in the PCV-2 evolution could have allowed explaining the phylogenetic relationship among strains without involving the presence of recombination events. However, a potential statistical power reduction in recombination detection cannot be excluded, both because of a higher complexity of the analyzed scenario and a more stringent multiple test correction.

Despite the efforts, it must be recognized that the presence in the final database of some, low fitness recombinant clusters cannot be completely excluded. In fact, the current methods are still perfectible, and recombination detection represents a partially unsolved challenge in viral phylogenesis. Moreover, the limited distance between parental strains and/or the progressive evolution after recombination occurrence, modifying the original recombinant sequence, could clearly complicate the detection of ancestral recombination events. Consequently, the confident identification of actual recombinant strains is probably far to be achieved. Nevertheless, the few potentially involved sequences and their limited practical role in the overall PCV-2 epidemiological scenario lessen the relevance of this formal shortcoming. Finally, apart from the theoretical knowledge on PCV-2 evolution, the presence of a highly divergent PCV2 cluster can be considered interesting *per se*, independently if originating through evolution or recombination.

The present study results confirm the well-known pattern of PCV-2 genotype natural history, characterized by an initial highest detection frequency of PCV-2a (Cluster3), which was outclassed by PCV-2b (Cluster11) and afterwards by PCV-2d (Cluster13). Differently from those major genotypes, the other ones showed a more restricted distribution. Unfortunately, the causes of the differential evolutionary fitness remain elusive and should deserve more focused investigations since they could contribute to the understanding of PCV-2 success (and potentially virulence) determinants.

Although the available sequence number approximately mirrors the overall PCV-2 genotypes population dynamics [14], it can hardly be considered and adequate proxy, being evident a relevant sampling bias due to the different sequencing intensity over time. This issue is particularly clear when combined with the concomitant location-based bias. For example, Asian available sequence number constantly rose over time. Thus, Asia became the most represented continent in the last 10 years, followed by North America, where a higher number of strains collected after 2011 was sequenced.

On the contrary, the amount of sequences provided by European countries is extremely limited, especially in recent years. Interestingly, PCV-2d (Cluster13) was the most commonly reported genotype in Asia, followed by North America, while its presence in Europe appeared low. Based on these evidences, it is hard to understand if the differences observed in the geographical distribution are actually due to a certain spatial clustering or can be mostly explained by sampling bias. The herein described scenario impedes any meaningful comparison among locations and, therefore, among the different applied control strategies, causing a lack of information that could be of pivotal importance for swine farming.

Similar conclusions can be drawn for the host distribution. In this case, most of the non *Sus scrofa domesticus* derived sequences originated from a limited number of countries. Therefore, even if an apparent over-representation of certain clusters was detected for wild boar, to confidentially establish if this association is due to actual biological reasons or to the sampling structure is almost impossible.

In conclusion, obtained results emphasize the limitations of the current PCV-2 molecular epidemiology knowledge and warrant the implementation of more effective monitoring measures. A unified classification scheme, proposed in the present study, surely represents a fundamental tool, establishing a “common language” among different research groups and diagnostic laboratories. At the same time, a huge effort of will must be set in place to guarantee a more organic and structured sampling activity and to encourage the sharing of properly annotated sequences in freely available databases.

## Supporting information

S1 Fig Complete dataset based phylogenetic tree. Neighbor-Joining phylogenetic tree reconstructed based on a complete collection of strains representative of the proposed PCV-2 clusters (color-coded). The bootstrap support has been displayed as a color-coded (from white (low) to black (high)) circle drawn at the corresponding node.
(PDF)

S2 Fig Mosaic plot depicting the relationship between Cluster and Host. The area of each cell is proportional to the count size. Cells have been color-coded and lines dotted based on standardized residuals (a standardized residual greater than 2 or lower than -2 is indicative of statistical significance). For graphical reasons, only the more relevant clusters and host categories have been plotted.
(PDF)

S3 Fig Workflow of genotype classification. A schematic workflow of the criteria to be used for strain classification and new genotype definition is provided.
(PDF)

S1 Table List of sequences belonging to each cluster and relative metadata. List of ORF2 sequences classified according to the respective cluster and genotype (when present). Additional metadata including collection date, host and continent are reported.
(XLSX)
