# Supplementary figures and images for: Porcine circovirus 2 (PCV-2) genotype update and proposal of a new genotyping methodology
Source: PLoS One. 2018 Dec 6;13(12):e0208585. doi: 10.1371/journal.pone.0208585 (PMC6283538; doi:10.1371/journal.pone.0208585)

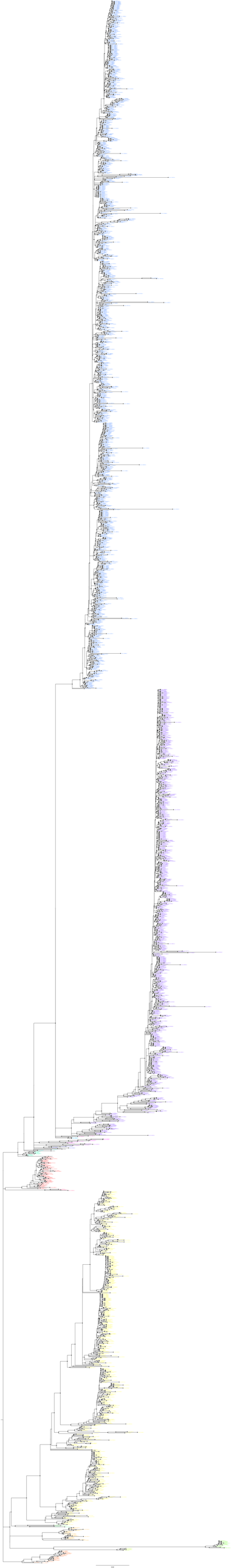

Supplement: S1 Fig — Neighbor-Joining phylogenetic tree reconstructed based on a complete collection of strains representative of the proposed PCV-2 clusters (color-coded). The bootstrap support has been displayed as a color-coded (from white (low) to black (high)) circle drawn at the corresponding node. (PDF) [file pone.0208585.s001.pdf]

# Genotype-Host association

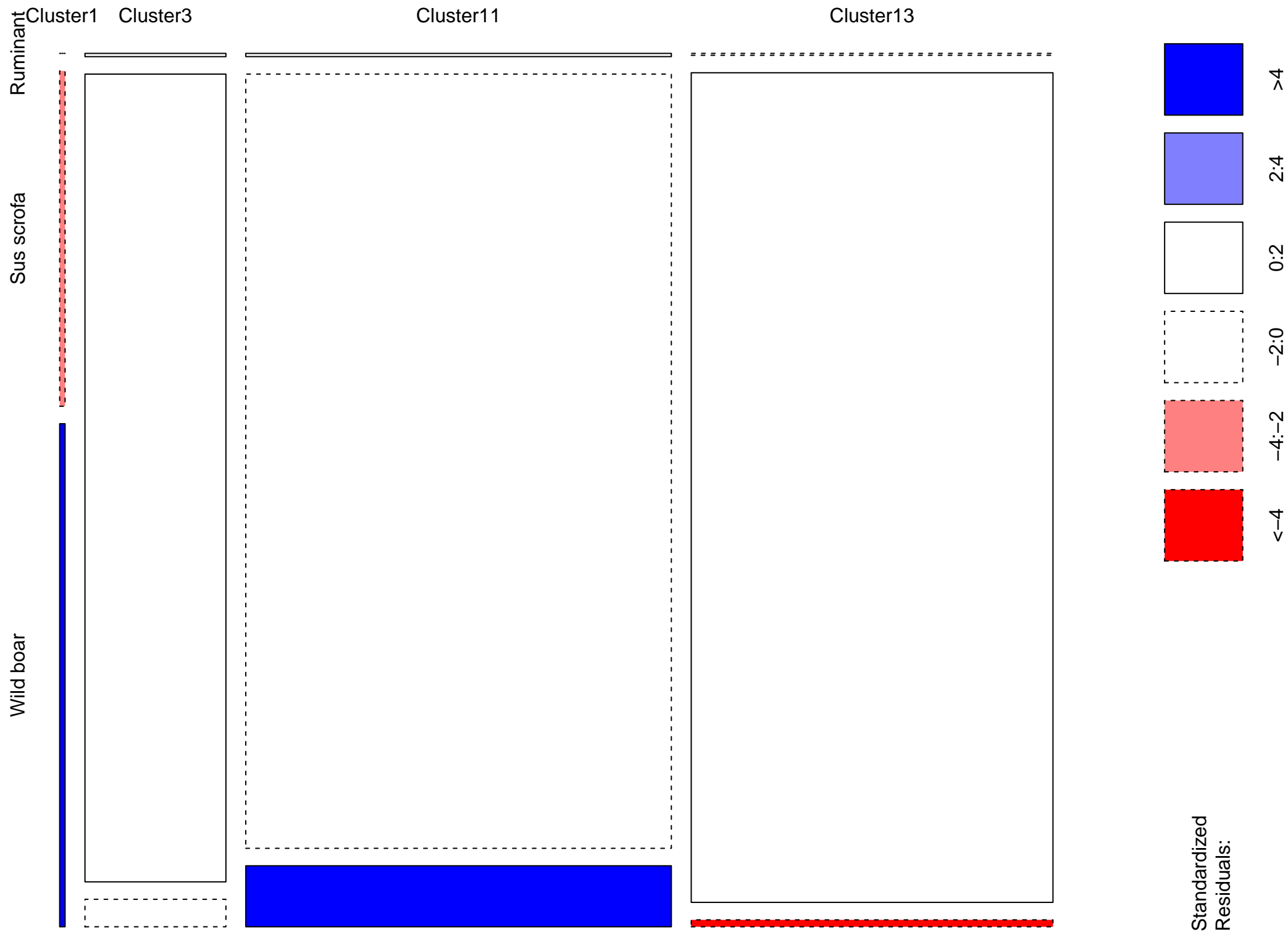

Supplement: S2 Fig — The area of each cell is proportional to the count size. Cells have been color-coded and lines dotted based on standardized residuals (a standardized residual greater than 2 or lower than -2 is indicative of statistical significance). For graphical reasons, only the more relevant clusters and host categories have been plotted. (PDF) [file pone.0208585.s002.pdf]
