# Supplementary material for: Porcine circovirus 2 (PCV-2) genotype update and proposal of a new genotyping methodology
Source: PLoS One. 2018 Dec 6;13(12):e0208585. doi: 10.1371/journal.pone.0208585 (PMC6283538; doi:10.1371/journal.pone.0208585)
Supplement: S3 Fig — A schematic workflow of the criteria to be used for strain classification and new genotype definition is provided. (PDF) [file pone.0208585.s003.pdf]

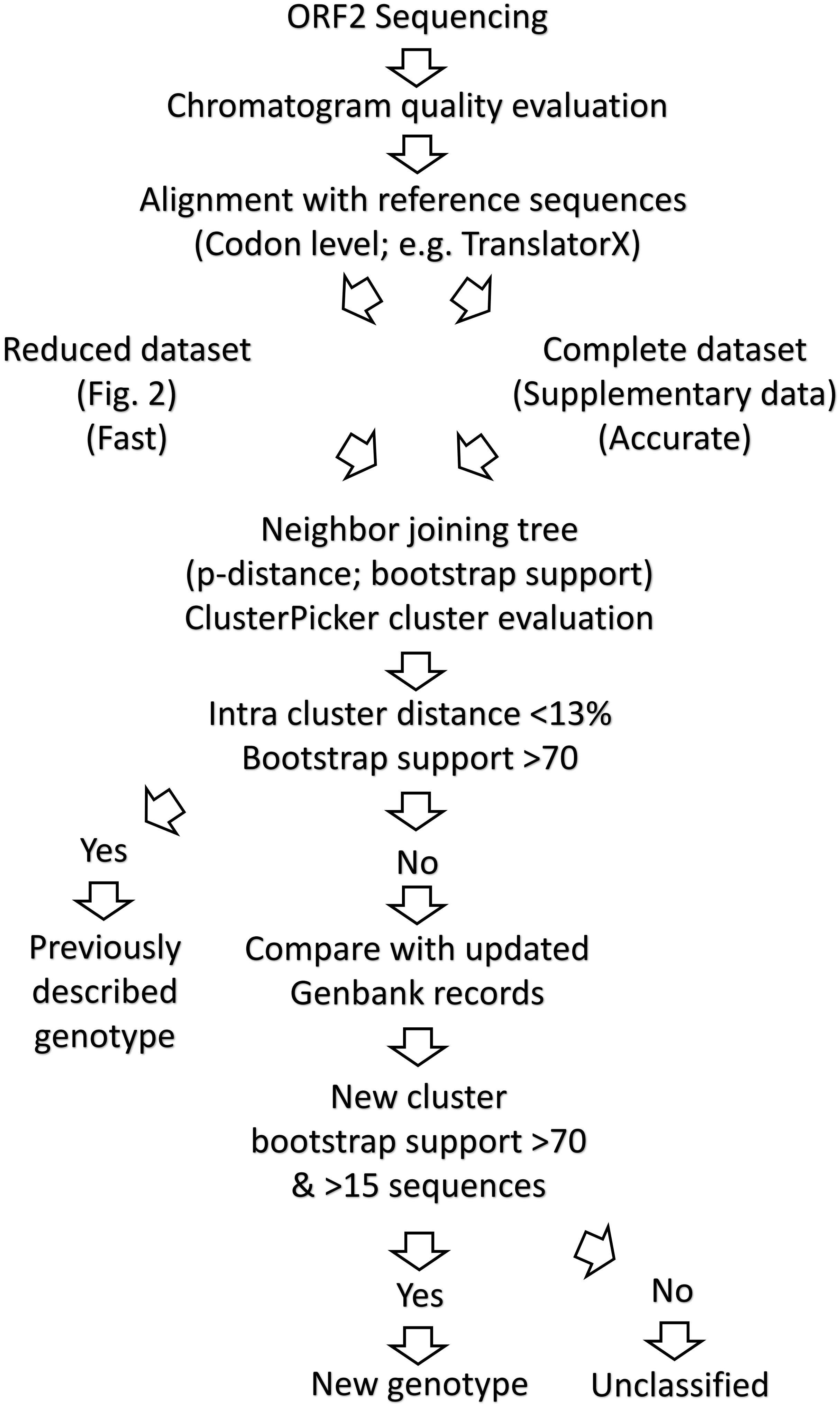

ORF2 Sequencing
Chromatogram quality evaluation
Alignment with reference sequences
(Codon level; e.g. TranslatorX)
Reduced dataset
(Fig. 2)
(Fast)
Complete dataset
(Supplementary data)
(Accurate)
Neighbor joining tree
(p-distance; bootstrap support)
ClusterPicker cluster evaluation
Intra cluster distance <13%
Bootstrap support >70
Yes
No
Previously
described
genotype
Compare with updated
Genbank records
New cluster
bootstrap support >70
& >15 sequences
Yes
No
New genotype
Unclassified
